# Book Review

**Published:** 1946

**Authors:** 


### Reviews of Books

**Industrial Toxicology.** By DONALD HUNTER, M.D., F.R.C.P. Pp. 80. London: Oxford University Press (Humphrey Milford).—Dr. Hunter's "Croonian Lectures" for 1942, which have already been published in the *Quarterly Journal of Medicine*, now appear in very handsome book form. These lectures give a detailed review, with full bibliography, of the effects of toxic substances encountered in industry. The information is of the greatest importance to industrial medical officers as well as to certifying surgeons and medical referees under the Workmen's Compensation Acts. Every practitioner will be glad to know where to turn to in cases of doubtful industrial diseases, from which his patients may be or may claim to be suffering. This is a work of great authority.

**Applied Physiology.** By SAMSON WRIGHT, M.D., F.R.C.P. Eighth Edition. Pp. xxx., 944. Illustrated. London: Oxford University Press. 1945. Price 30s. net.—In the twenty years which have elapsed since its first appearance, "Samson Wright" has earned an established place among the standard textbooks. Of great value to the pre-clinical and clinical student, and well-nigh indispensable to the post-graduate it has, alas, increased in size with its years. With this edition it has added 150 pages to its stature together with a considerable number of new illustrations. The author has been concerned lest many of his readers pay too little attention to the illustrations. In an attempt to remedy this an appendix on the critical analysis of figures has been added. The student who studies this carefully will not only gain much when reading this book, but whenever confronted with similar figures or tracings. Needless to say, the work has been brought completely up to date by the addition of new sections and the rewriting of many more. As an illustration of the thoroughness with which this has been done may be cited the photograph illustrating *urticaria factitia*, which consists of the picture of a member of the British Merchant Navy across whose broad back are whealed the words "There'll always be an England."

**Class-Book of Practical Embryology.** By P. N. B. Odgers. Pp. 63. Illustrated. London: Oxford University Press. 1945. 7s. 6d. net.—This is a small but very useful class-book of practical embryology. It is based upon 12 transverse sections through a 6 mm. pig embryo, and 13 through a 10 mm. pig embryo. Representative sections are clearly drawn, and briefly described. Some simple orienting diagrams are also included. Within the limits of what it sets out to achieve, this manual of practical embryology for medical students is excellent, and can be thoroughly recommended.

**ABC of Medical Treatment.** By E. NOBLE CHAMBERLAIN, M.D., M.Sc., F.R.C.P. Pp. viii., 206. London: Oxford University Press. 1946. Price 10s. 6d. net.—In this handy little book the subject matter has been arranged alphabetically, and indicates briefly and succintly the main lines of treatment available for any given disease. There is a short appendix on the use of Penicillin and its indications. It is a pity that in some places the instruction given lacks precision as, for instance, the recommendations that pneumococcal arthritis should be treated by "sulphonamides" and hæmophilia by "snake venom." It is clearly important that a suitable sulphonamide should be used and the right sort of snake venom. However, intended as it is for the general practitioner wishing to obtain ready reference to the essentials of treatment, it should prove of great help. By no means the least valuable part is a chapter by Miss Simmonds on Diets. This includes detailed diet sheets suitable for a large variety of conditions and these are, in most cases, presented as "war-time" and "post-war" alternatives.

**Textbook of the Practice of Medicine.** Edited by F. W. PRICE, M.D., C.M., F.R.C.P., F.R.S. Seventh Edition. Pp. xlvi., 2034. Illustrated. London: Oxford University Press (Geoffrey Cumberlege). 1946. Price 42s. net.—In the preface to the first edition of this work which appeared in 1922, the editor expressed the hope "that the book may be considered a credit to the London School of Medicine." The rapid succession of impressions and new editions leaves little room for doubt that this hope has been fully realized. Covering as it does the whole field of medicine and written by a most distinguished band of contributors, Price's *Medicine* may well claim to present British Medicine to the world. In this edition many sections have been re-written and new sections added, so that the many contributions which the war years have made to medicine have all been included. It can be recommended confidently to all senior students as a textbook, and to practitioners as an invaluable book of reference.

**Medical Aspects of Growing Old.** By A. T. TODD, M.B., M.R.C.P. Pp. vi., 164. Illustrated. Bristol: John Wright & Sons Ltd. 1946. Price 15s. net.—It has been said that it is "a social duty gracefully to grow old," so that there will be many who will welcome the practical suggestions that Dr. Todd has made in this book. He says that he has written it in an "attempt to lessen the dependance of the elderly person, and help him to become useful and happy, and also to defer the onset of old age if a start is made in middle age." Accordingly, it is phrased so that the layman can read it. The scheme is the consideration of the whole metabolic processes—but this has involved the inclusion of original ideas presented without much evidence for their acceptance. Thus it needs to be perused with discrimination. For instance, it is stated that fats are of no metabolic value in the dietary, though partly beneficial as appetizers. Milk and cheese are eschewed, but butter is permissible. It is difficult to appraise a volume with a dual purpose and the lay reader may find it difficult to comprehend some unusual views on circulating and respiratory disturbances. Perhaps the most useful section is that on retirement, although those on the care of the skin, scalp and feet are helpful. The prostatic enlargement and chronic vulvitis are discussed in detail. On the whole, the book is stimulating and suggestive. The mental attitude to old age might with advantage have been considered for, after all, so long as one remains "young in thought," the years pass unnoticed. There are some useful tables on food values and vitamins, but the index is far from comprehensive.

**A Synopsis of Forensic Medicine and Toxicology.** By E. W. CARYL THOMAS, M.D., B.Sc., D.P.H., Barrister-at-Law. Second Edition. Pp. 179. Bristol: John Wright & Sons Ltd. 1945. Price 10s. net.—It is generally conceded that it is not necessary for a medical practitioner to acquire an expert knowledge of forensic medicine, but that it is essential to be able to deal promptly with forensic problems which may occur in everyday practice. These include the signing of numerous statutory certificates, the giving of evidence in Courts of Law, the methods of dealing with mentally disordered persons, and a considerable number of other similar duties. These are dealt with in a most admirable way by Dr. Caryl Thomas in his *Synopsis of Forensic Medicine and Toxicology*, which has been compiled with great care and accuracy. It is gratifying to notice that the author has treated the Matrimonial Causes Act, 1937, in some detail, since at the present time medical evidence is more frequently called for in the Divorce Court than in normal years. The subject of Toxicology has been dealt with in sufficient detail for students to gain some knowledge of the elementary principles involved in toxicological examinations.

**The Psycho-Pathology of Prostitution.** By EDWARD GLOVER, M.D. Pp. 16. London: Institute for the Scientific Treatment of Delinquency. 1945. Price 1s.—This short pamphlet, 16 pages, is an exposition of factors supposedly responsible for prostitution considered from the psycho-analytic viewpoint. It does not claim to provide answers to the practical problems, nor can it be expected to supply useful data. The theoretical reasoning, as far as it goes, is sound, but is biased by the narrow view point. Many people would lay more stress on the backwardness of the prostitute than does the author. To psycho-analysts the paper is an interesting essay, but the approach is neither instructive nor useful to others.

**Neurosis and the Mental Health Services.** By C. P. BLACKER, M.D., F.R.C.P. Pp. xxii., 218. London: Oxford University Press (Geoffrey Cumberlege). 1946. Price 21s. net.—This comprehensive and detailed report is based on the facts found by the Regional Surveys of 1942–43. Full statistics regarding number of clinics, type of accommodation, patients seen and work done at the various centres, are given. The question as to whether neurosis is on the increase is discussed. The second and third parts deal with long-term and short-term policy respectively. The latter is primarily one of training an adequate number of psychiatrists, psychologists and psychiatric social workers. Bristol is envisaged as being one of the leading post-graduate training centres outside London. Long-term policy involves a big increase in the psychiatric services and the requirements of a population of a million people are discussed in detail. A hundred beds outside the present Mental Hospitals will be required. In university centres these beds would be part of the Teaching Psychiatric Unit which should if possible be adjacent to and form part of the main teaching hospital. A considerable expansion of out-patient work will be needed. Six new patients or four new patients and four treatment cases are regarded as the maximum for a single session. Work with children is given first priority, and an area with a population of a million would require ten Child Guidance Centres and three Clinics, also a Hostel for fifty unstable or difficult children. This report is an outstanding piece of work, and Sir William Jameson's comment in the introduction that " the reactions it evokes will be taken into account when framing future policy " should not be overlooked.

**Outlines of Physical Methods in Medicine.** By G. D. KERSLEY, M.D., F.R.C.P. Pp. ix., 85. Illustrated. London: William Heinemann Medical Books Ltd. 1945. Price 6s. net.—Dr. Kersley, in his preface, says that this little book was produced from notes for lectures to medical officers undergoing training in Physical Medicine, and to R.A.M.C. masseurs. It is quite impossible to condense the whole of physical medicine into eighty pages, especially when a chapter on X-rays is included! Medical students, who want to get a preliminary idea of what Physical Medicine is all about, will find this book useful. There is an extraordinary diagram of the faradic coil on plate 3. The book is well printed and reasonably priced.

**Cellona Technique :** a handbook to the Functional Treatment of Fractures, with section on Methods of Plaster Fixation in Tuberculous Disease of the Spine and Hip Joints. Sixth Edition. Pp. 105. Illustrated. Hull: T. J. Smith & Nephew Ltd. 1945.—This useful little volume has been enlarged by 50 per cent. and completely revised, and is a great credit to its producers, Messrs. T. J. Smith & Nephew. As always, it shows the hand of the orthopædic specialists throughout its pages. Essentially practical in every section and in all the numerous illustrations, it is a very useful book for all members of the Medical and Nursing professions and also for plaster orderlies. There is little one can criticize in the way it handles the scope of plaster technique and the advantages of Cellona bandage. The illustrations of the scaphoid plaster with the adducted and unopposed thumb is, however, a frequent error. The thumb should be opposed and adducted, thus improving the fixation and vastly enhancing the performance of the hand. The " Tobruk " Plaster, demonstrated on page 66, was superseded later in the war by a later model involving less risks and more adapted to inspection of the limb. The quality of paper and production has not suffered, although this was a wartime production. It is certainly a book which should be studied by all who work within the ever-growing scope of plaster of paris.

**Cookery Book for Diabetics.** By The Diabetic Association. Pp. 82. London: H. K. Lewis & Co. Ltd. 1945. Price 4s.—This book almost makes one feel that a diabetic in the home might be a help if it led to the use of the recipes here set forth. It really is an excellent production, and Professor Mottram's words of introduction seem the most appropriate way of summing up one's own feelings. The hints for diabetic cookery are particularly attractive, they throw out so much in the way of challenge and common sense.

